# Online dating: predictors of problematic tinder use

**DOI:** 10.1186/s40359-024-01566-3

**Published:** 2024-02-29

**Authors:** Germano Vera Cruz, Elias Aboujaoude, Lucien Rochat, Francesco Bianchi-Demicheli, Yasser Khazaal

**Affiliations:** 1 Department of Psychology, CRP-CPO, University of Picardie Jules Verne, Amiens, UR 7273 France; 2 Department of Psychiatry and Behavioral Sciences, Stanford University School of Medicine, Stanford, CA USA; 3 Cedars-Sinai Medical Center, Los Angeles, CA USA; 4 Addiction Division, Department of Psychiatry, University Hospitals of Geneva, Geneva, Switzerland; 5 Department of Obstetrics and Gynecology, University Hospitals of Lausanne, Lausanne, Switzerland; 6 Center for Preventive & Integrative Medicine, Clinique des Grangettes and Center for Internal Medicine and its Specialties, Clinique La Colline, Hirslanden Group, Geneva, Switzerland; 7 Addiction Medicine, Department of Psychiatry, Lausanne University Hospital, Lausanne, Switzerland; 8 Research Centre, University Institute of Mental Health at Montreal and Department of Psychiatry and Addiction Montreal University, Montreal, Canada

**Keywords:** Dating apps, Problematic internet use, Tinder, Gaming disorder

## Abstract

**Background:**

Geolocation apps have radically transformed dating practices around the world, with profound sociocultural implications. Few studies, however, have explored their addictive potential or factors that are associated with their misuse.

**Objective:**

The present study aimed to assess the level of problematic Tinder use (PTU) in an adult sample, using a machine learning algorithm to determine, among 29 relevant variables, the most important predictors of PTU.

**Methods:**

1,387 users of Tinder (18–74 years-old; male = 50.3%; female = 49.1%) completed an online questionnaire, and a machine learning tool was used to analyze their responses.

**Results:**

On 5-point scale, participants’ mean PTU score was 1.91 (SD = 0.70), indicating a relatively low overall level of problematic app use. Among the most important predictors of Problematic use were the use of Tinder for enhancement (reduce boredom and increase positive emotions), coping with psychological problems, and increasing social connectedness. The number of “matches” (when two users show mutual interest), the number of online contacts on Tinder, and the number of resulting offline dates were also among the top predictors of PTU. Depressive mood and loneliness were among the middle-ranked predictors of PTU.

**Conclusion:**

In accordance with the Interaction of Person-Affect-Cognition-Execution model of problematic internet use, the results suggest that PTU relates to how individual experience on the app interacts with dispositional and situational characteristics. However, variables that seemed to relate to PTU, including lack of self-esteem, negative mood states and loneliness, are not problems that online dating services as currently designed can be expected to resolve. This argues for increased digital services to identify and address potential problems helping drive the popularity of dating apps.

## Introduction

Data from Western Europe and North America [1–3] suggest that the majority of new intimate partner relationships now begin online, over a dating app or website. Indeed, the last decade has witnessed a dramatic shift in how relationships now form—away from bars, restaurants, and one’s family, friends or professional circles, and toward online and digital environments [3].

The main reasons for the migration away from “traditional” ways include hesitation to convey interest in a potential mate face-to-face due to fear of rejection, self-consciousness, concern about bothering the other person or, in the case of sexual minorities, uncertainty of the sexual preferences of the other person and fear of stigma [2, 4, 5]. Other reasons have to do with the built-in characteristics of the dating apps or websites themselves, such as the access they provide to a potentially unlimited number of mates; their ease of use; the minimal effort required to express interest or lack thereof (e.g., swipe right or left); anonymity, particularly in non-heteronormative relationships; immediacy, which allows for greater spontaneity, directness and immediate psychological rewards [6]; and affordability [7–9]. However, features such as unlimited access, anonymity and immediate reward, which allow people to more easily find intimate partners online, can also encourage impulsivity and increase users’ risk for developing problematic use [7, 8, 10–12].

Among technology-supported dating platforms, smartphone apps are the most popular [3]. Recent studies suggest that people’s main motives for using them include: finding committed romantic partners; finding casual sex partners; boosting self-esteem; entertainment; relief from boredom; social interaction; identity exploration; and curiosity [8, 12–16]. Women are more likely to use dating apps to find committed love and improve self-esteem, whereas men are more likely to use them to find casual sex. Furthermore, compared to younger users, older ones are slightly more likely to use dating apps to find casual sex partners [8].

Regarding the problematic use (addictive-like use)^1^ of geolocation dating apps, a previous study of predictors [8] showed that self-esteem enhancement, casual sex and boredom were among the strongest. A systematic review by Bonilla-Zorita et al. [17] suggested that the personality correlates of neuroticism, sociability, sensation-seeking and sexual permissiveness were related to greater use of online dating services and that sex searches and self-esteem enhancement, in particular, were significant predictors of problematic use.

While the previous studies on the problematic use of dating app have yielded some interesting results, most have been relatively small (< 500 participants) and have explored a rather narrow set of potential predictors relating mostly to personality traits and sociodemographic characteristics. For example, our group conducted a systematic search of articles using the PsycINFO and Web of Science databases (January 2024) with the following keywords: “online dating problematic use”; “dating app problematic use”; and “Tinder problematic use”. A total of 33 articles were retrieved, 7 of which were quantitative studies reporting the relationships between a set of predictor variables and problematic use of online dating services as outcome. The number of predictor variables in each of the 7 studies ranged from 2 to 5. The most represented predictor variables were: motives to use online dating services, impulsivity, personality traits, well-being, social anxiety, loneliness and self-desirability [17]. The number of participants ranged from 269 [18] to 430 [7].

To more fully understand the factors associated with the problematic use of dating apps, large, representative studies that assess a relatively large number of variables related to participants’ specific behaviors and interactions with the app, as well as person-specific variables, seem crucial.

### Study purpose

The present study aimed to: (a) assess the level of problematic dating app use among users of the dating app Tinder; (b) examine the bivariate relationships between the 29 variables (including socio-demographic information, person-specific factors [also called individual factors], motive measures, behavioral measures on the dating app use and app satisfaction indices) and problematic Tinder use, (c) explore the multivariate relationships, using a machine learning algorithm to determine the most important predictors of problematic use from the 29 aforementioned variables. Given that it is the most popular worldwide [3, 19] and can therefore potentially yield more applicable results, the dating app Tinder was chosen for this study.

We performed both bivariate (correlation) and multivariate (multiple regression) analyses because they yield different information about the relationship between predictor variables and the outcome variable [20]. Bivariate correlations (as well as bivariate regression) inform us about the ‘singular’ relationships of each predictor with the outcome, ignoring other predictors. Multivariate regressions, on the other hand, inform us about the relationship between each predictor and the outcome, independently of other predictors in the model, thus controlling for ‘confounds’ (the risk of attributing an effect to one variable when it might be due to another). Consequently, bivariate and multivariate results for a given predictor do not always agree. Thus, conducting both types of analysis is a strategy that is more likely to unveil complex relationships between predictors and outcomes [20]. For instance, in the current study, the machine learning multivariate analysis was designed to give the specific contribution of each independent variable in the overall prediction model (which is indispensable to rank-order them), while controlling for multicollinearity and compound effects [21].

In the present study, the inclusion of sociodemographic variables (e.g., age, sex, sexual orientation), person-specific variables (e.g., mental health-related measure [depression], self-esteem, loneliness, attachment style, sexual desire), motives for using the dating app variables (e.g., enhancement, coping, increase social connectedness), behavioral measures relative to the use of the dating app (e.g., use duration, patterns of use related variables) and satisfaction with the service used variables was determined as a function of the Interaction of Person-Affect-Cognition-Execution model (I-PACE) of problematic Interned-based services use [22]. The I-PACE posits that ‘addictive’ use of Internet-based services is the consequence of interactions between person-specific factors (represented in the current study by sociodemographic and person-specific variables), perception of the situation (in the current study, no variable represented this dimension), affect and cognitive responses (represented in the current study by the motives to use the service and the impulsivity variables), decision to use a certain service in a certain way (represented in the current study by variables related to participants’ behavior on Tinder) and gratification and/or compensation (represented in the current study by variables related to the satisfaction with Tinder use). Other variables that would have been suitable for this study, such as personality traits, were not captured in the collected data.

## Methods

Secondary data from Rochat et al. [23] was used.

### Participants

A total of 1,387 Tinder-using subjects (Male = 698[50.3%], Female = 681[49.1%], Non-binary = 8[0.6%] who completed the online questionnaire were included in the present study. Participants were between 18 and 74 years old (M = 29.41, SD = 8.98) and were English speakers. Information on their countries of residence was not collected. It must be noted that in the study by Rochat et al. (1,159 participants) [18], the non-heterosexual participants (228) were excluded, which explains the difference on the number of participants between the present study (1,387) and the previous conducted based in the same data.

### Recruitment and sampling procedures

Recruitment for the original larger study on online dating [23] was carried out through advertisements on social media (e.g., Facebook) and relevant Internet forums and websites. English-speaking Tinder users over 18 years of age were invited to participate, making this a non-random sample. Participants gave informed consent before being able to view study questions. Responses were anonymous, and no identifying participant information was stored.

### Ethics

The study is part of a larger study on online dating and was carried out in accordance with the Declaration of Helsinki [24] and approved by the Ethical Committee of Swiss Human Research Act (Ethical approval GE 12–165). Study participants gave informed consent online before anonymously completing the assessments via a SurveyMonkey link. Responses were transmitted over a Secure Socket Layer-encrypted connection.

### The study predictor variables

A total of 29 predictor variables were assessed.

*Participants’ socio-demographic characteristics (4 variables)*. These characteristics consisted of age, sex, sexual orientation, and marital status.

*Participants’ Tinder use patterns (8 variables).* These included: whether the participant was looking for online Tinder contacts that can lead to offline contacts (0 = No, 1 = Yes); the number of months using Tinder (range: 1 [less than 3 months] to 5 [more than 2 years]); looking for uncommitted sex partners (range: 1 [not true at all] to 7 [absolutely true]); looking for committed romantic partners (range: 1 [not true at all] to 7 [absolutely true]); the number of Tinder-initiated online and offline contacts in the preceding 6 months (range: 1 [0 person] to 8 [more than 50 persons]); the participant’s “liking” behavior (conceived as a measure of partner selectiveness) (range: 1 [“I give as many as I can”] to 5 [“I give only to the profiles where I like the pics and the description”]; and the number of current “matches” on the app (when two users show mutual interest by using the app’s “swipe” functionality; on Tinder, a “match” is required for two users to able to contact each other).

*Participants’ level of satisfaction (2 variables).* This included two items: the participant’s satisfaction with Tinder use (range: 1 [not at all] to 4 [entirely yes]); and the participant’s satisfaction with Tinder offline meetings (actual “dates”) (range: 1 [not at all] to 4 [definitely yes]). For each participant, two scores were calculated: the satisfaction with Tinder use score and the satisfaction with the Tinder offline dates score. Higher scores reflect greater satisfaction.

*Participants’ mood (1 variable).* Focusing specifically on depressed mood, this was measured using the Short Happiness and Depression Scale (SDHS) [25], which includes six items assessing happiness (e.g., “I feel happy”) or depression (e.g., “I feel dissatisfied with my life”). For each item, the response ranges from 1 (never) to 4 (often). In the current study, the scoring of the happiness items was reversed to ensure that higher scores indicate depressive mood, and lower scores indicate happiness. The scale’s Cronbach α in the current study was 0.73.

*Participants’ level of loneliness (1 variable).* This was measured using a single item [26] (“Overall I am feeling lonely”), with a response scale ranging from 1 (I disagree) to 5 (I totally agree). Higher scores indicate greater loneliness.

*Participants’ self-esteem (1 variable).* This was measured using the Single-Item Self-Esteem Scale (SISE) [27]. The single item (“I have high self-esteem”) was rated on a 5-point Likert scale, ranging from 1 (not very true for me) to 5 (very true for me). Higher scores indicate greater self-esteem.

*Participants’ sexual desire (2 dimensions = 2 variables).* This was measured using the Sexual Desire Inventory (SDI**)** [28, 29], which consists of 14 items assessing two dimensions of sexual desire: *solitary* (i.e., the desire to engage in sexual behavior alone) and *dyadic* (i.e., the desire to engage in sexual activity with another person). Using a Likert response scale, participants measured each of the two dimensions of each item on: frequency from 0 (not at all) to 7 (more than once a day); intensity from 0 (no desire) to 8 (strong desire); and importance from 0 (not at all important) to 8 (extremely important). For each participant, two scores were calculated: the dyadic sexual desire score and the solitary sexual desire score. Higher scores indicate greater sexual desire in each of the two dimensions. The subscales’ Cronbach α values were 0.78 and 0.68, respectively.

*Participants’ motives for using Tinder (3 dimensions = 3 variables).* This was assessed using the Cybersex Motives Questionnaire (CMQ) [30], adapted for this study to address Tinder use only. The CMQ consists of 14 items that assess three possible cybersex motives: *enhancement* (to increase positive emotions, e.g., “to be entertained”); *coping* (strategies that reduce depressive mood, e.g., “to forget my problems”); and *social* (a desire to increase social connectedness, e.g., “because I need to socialize with others”). A 5-point Likert response scale ranging from 1 (never) to 5 (always or almost always) was used to measure responses. Thus, for each participant, three scores were calculated: an enhancement motive score, a coping motive score, and a social motive score. Higher scores reflect greater endorsement of the specific motive for using Tinder. The subscales’ Cronbach α values were 0.77, 0.84 and 0.75, respectively.

*Participants’ attachment style (2 dimensions = 2 variables).* This was assessed using the Experiences in Close Relationships – Revised [31] questionnaire, which includes 36 items designed to assess anxious attachment (i.e., the extent to which people are insecure vs. secure about the availability and responsiveness of romantic partners) and avoidant attachment (i.e., the extent to which people are uncomfortable being close to others vs. secure depending on others). Associated with each item is a 7-point response scale, ranging from 1 (disagree strongly) to 7 (agree strongly). For each participant, two scores were calculated: anxious attachment style score and avoidant attachment style score, with higher scores indicating a greater anxious or avoidant attachment style, respectively. The subscales’ Cronbach α values were 0.73 and 0.71, respectively.

*Participants’ level of impulsivity (5 dimensions = 5 variables).* This was measured using the Short UPPS-P Impulsive Behavior Scale [32]. UPPS-P stands for Urgency, Premeditation (lack of), Perseverance (lack of), Sensation Seeking and Positive Urgency. It includes 20 items that assess five facets of impulsivity: *positive urgency* (e.g., “When I’m happy, I often can’t stop myself from going overboard”), *negative urgency* (e.g., “When I feel rejected, I often say things that I later regret”), *perseverance* (lack of) (e.g., “I am a person who always gets the job done”), *premeditation* (lack of) (e.g., “I usually make up my mind through careful reasoning”), and *sensation-seeking* (e.g., “I welcome new and exciting experiences, even if they are a little frightening or unconventional”). Associated with each item was a 4-point response scale, ranging from 1 (I agree strongly) to 4 (I disagree strongly). Thus, for each participant, five scores were calculated: positive urgency impulsivity score, negative urgency impulsivity score, lack of perseverance impulsivity score, lack of premeditation impulsivity score, and sensation-seeking impulsivity score. Higher scores indicated greater impulsivity. The subscales’ Cronbach α values were 0.80, 0.82, 0.76, 0.67, and 0.70, respectively.

### The study outcome

*Participants’ level of problematic Tinder use (1 variable).* This was measured using the Problematic Tinder Use Scale (PTUS) [7]. This instrument consists of 6 items (e.g., during the last year how often have you tried to cut down on Tinder use without success?), modeled on Griffiths’ [33] six-component addiction framework and measuring *salience*, *tolerance*, *mood modification*, *relapse*, *withdrawal*, and *conflict* as they pertain to participants’ use of Tinder. Associated with each item was a 5-point Likert response scale, ranging from 1 (never) to 5 (always). For each participant, one overall PTUS score was calculated, with higher scores suggesting greater addictive use. The scales’ Cronbach α was 0.77.

All scales utilized for the predictor variables and outcome measure have been validated (see references associated with each).

### Data analysis

The 29 predictor variables and the one outcome measure were used in three different models of data analysis.

First, we conducted a descriptive data analysis (means [M], standard deviations [SD] and frequency computations) using the SPSS statistical software (version 28).

Second, we conducted bivariate correlations analysis between the 29 predictor variables and the outcome variable, SPSS statistical software (version 28). The four categorical non-ordered predictor variables, also referred to as *nominal variables* (sex, marital status, sexual orientation, looking for Tinder online contacts that can lead to offline contacts), were included in an analysis of variance (ANOVA) that examined their effects on participants’ problematic Tinder use (PTU) and yield the follow-up Tukey post-hoc comparison tests.

Third, we built the best machine learning regression model possible (with all 29 independent variables as predictors of the outcome [PTU]) to rank-order the predictors from the most important to the least important. In this task, we used the machine learning Random Forest algorithm (“randomForest” R package) [21]. Random Forest (RF) regression models help quantify, among other outputs, the importance of each predictor on the basis of a measure called %IncMSE (per cent increase in mean squared error). The %IncMSE expresses the increase in MSE (estimated with out-of-bag cross validation) as a result of variable j being permuted (values randomly shuffled). In other words, it describes how much (in terms of percentage) the MSE increases by excluding each variable. The more the MSE increases, the more important the variable is for the successful prediction. Thus, variables can be presented in ranked order of importance (Table 3). For more information on the RF algorithm function, see Breiman [21]. Still, it must be noted that machine-learning classification and regression algorithms do not make inference statistics; this explains why we used standard statistical methods to obtain inference information.

We used machine learning algorithms rather than standard statistical methods because its hyperparameters allow us to build and test different models in terms of prediction capabilities and to choose the best prediction models as function of specific metrics [21]. Furthermore, unlike standard linear regression models, machine learning algorithms are nonparametric—i.e., they do not impose a particular structure on the data. As such, they can capture nonlinear relationships, including interactions among the all modeled predictor variables. As matter of fact, the algorithm we used is considered among the best for the prediction and rank-ordering of the most important predictor variables [21, 34–36]. Compared with traditional regression, RF is considered robust for high-dimensional data scenarios, due to its ensemble nature (separately bootstrapping thousands of decision trees, then averaging their results).

Finally, machine learning models are designed for prediction. They are built in two phases [21]: the learning phase where the model analyzes and “learn” from the variables relations/associations; and the second phase where the model uses the “learned knowledge” to predict. In the present study, the dataset was split as follows: train-set = 70% of the sample; test-set = 30%. The selected model had the following parameters: “ntree”=500, meaning that each RF model was constructed from 500 regression trees. We left “mtry,” the number of predictors available for splitting at each tree node, at its default value (one-third of the total number of predictors). We selected the model with performance metrics indicating low overfitting, while having the highest explained variance and the lowest residual error in the test-set. Indeed, the selected model predicted a majority of the variance in the outcome variable (R^2^ = 58%), with very low residual error (RMSE = .19).

The data had no missing values. Thus, the entire 1,387 participants were included in all statistical analyses presented above.

## Results

### Descriptive statistics

Table 1 shows the descriptive statistics of the 29 predictor variables and the outcome variable.

As shown in Table 1, participants’ mean age and standard deviation (M = 29.41, SD = 8.98) suggest that the age distribution is diversified among the adult population (18–74 years-old). Also, male and female participants (50.3% and 49.1% respectively) were almost equally represented. Interestingly, 65.3% of participants were “in a relationship” or married, the remaining were single. The large majority of participants (84.1%) were heterosexual, and almost half of participants had been using Tinder with the goal of finding someone they could meet offline.

**Table 1:** Descriptive statistics related to the variables used in the present study

Variables	Scale	Mean/Frequency	SD
Age	18–74	29.41	8.98
Looking for Tinder online contacts that can lead to offline contacts*		Non = 689(49.7%) Yes = 698(50.3%)	
On Tinder looking for committed romantic partner	1–7	3.28	1.94
On Tinder looking for uncommitted sex partner	1–7	3.48	2.01
Number of “Matches”	0–2500	39.45	134.42
Number of months using Tinder	1–5	2.53	1.49
Score of partner selectiveness	1–4	3.29	1.04
Number of online contacts	1–8	3.41	1.87
Number of offline contacts	1–8	2.00	1.26
Satisfaction with Tinder use	1–4	2.39	0.79
Satisfaction with Tinder offline dates	1–4	2.44	1.13
Enhancement motive to use Tinder	1–5	2.66	0.79
Coping motive to use Tinder	1–5	2.17	0.97
Social motive to use Tinder	1–5	2.67	0.92
Anxious attachment style	1–7	3.81	1.28
Avoidant attachment style	1–7	3.19	1.08
Dyadic sexual desire	1–9	5.74	1.54
Solitary sexual desire	1–9	4.87	2.07
Negative urgency impulsivity	1–4	2.61	0.69
Positive urgency impulsivity	1–4	2.65	0.59
Lack of premeditation impulsivity	1–4	1.87	0.53
Lack of perseverance impulsivity	1–4	1.96	0.56
Sensation seeking impulsivity	1–4	2.73	0.62
Depressive mood	1–4	2.22	0.66
Score of loneliness	1–5	2.89	1.29
Self-esteem	1–4	2.37	0.84
Problematic Tinder use**	1–5	1.91	0.70

N = Number of participants

*Nominal variables (that is categorical non-ordered variables); **Outcome variable; SD = standard deviation

For 14 of the 25 categorical-ordered and continuous variables assessed, participants’ mean scores were above the midpoint of the used scale. The 14 predictor variables were: number of months using Tinder; satisfaction with Tinder; satisfaction with Tinder offline dates; the mean score of partner selectiveness; enhancement motive to use Tinder; anxious attachment style; social motive; dyadic sexual desire; solitary sexual desire; negative urgency impulsivity; positive urgency impulsivity; sensation seeking impulsivity; loneliness; depressive mood; and the mean score of self-esteem.

The mean score of PTU (the outcome variable) was 1.91 (SD = 0.70) on a 5-point scale.

### Bivariate relationships (correlation and ANOVA statistics)

Table 2 displays the bivariate correlation statistics between the predictor variables and the outcome variable. To interpret the *r* values, it must be considered that [37]: very high correlations range from 0.90 to 1.00 (-0.70 to -1.00); high correlations range from 0.70 to 0.90 (-0.70 to − 0.90); moderate correlations range from 30 to 0.70 (-0.30 to − 0.70); low correlations range from 0.20 to 0.30 (-0.20 to − 0.30); negligible correlations range from 0.00 to 0.20 (0.00 to − 0.20).

**Table 2:** Bivariate correlations between the 25 categorical ordered/continuous independent variables and the participants’ Tinder problematic use

Variable categories / variables	*r*	95% CI	
		Lower CI	Upper CI
Age	− 0.067	− 0.119	− 0.014
On Tinder looking for committed romantic partner	0.351	0.304	0.397
On Tinder looking for uncommitted sex partner	0.402	0.357	0.445
Number of “Matches”	0.072	0.020	0.125
Number of months using Tinder	0.050	− 0.003	0.102
Partner selectiveness on Tinder	− 0.271	− 0.319	− 0.222
Number of online contacts	0.440	0.396	0.481
Number of offline contacts	0.480	0.438	0.519
Satisfaction with Tinder use	0.345	0.297	0.390
Satisfaction with Tinder offline dates	− 0.074	− 0.126	− 0.022
Enhancement motive to use Tinder	0.554	0.516	0.589
Coping motive to use Tinder	0.623	0.589	0.654
Social motive to use Tinder	0.532	0.493	0.568
Anxious attachment style	0.305	0.256	0.352
Avoidant attachment style	0.166	0.114	0.217
Dyadic sexual desire	0.218	0.167	0.267
Solitary sexual desire	0.235	0.185	0.285
Negative urgency impulsivity	0.175	0.124	0.226
Positive urgency impulsivity	0.232	0.182	0.281
Lack of premeditation impulsivity	0.102	0.050	0.154
Lack of perseverance impulsivity	0.046	− 0.006	0.099
Sensation seeking impulsivity	0.171	0.120	0.222
Depressive mood	0.089	0.036	0.141
Loneliness	0.299	0.251	0.346
Self-esteem	0.077	0.024	0.129

N = number of participants, *r* = correlation coefficient; CI = confidence interval

As shown on this table, none of the predictor variables are *highly* or *very highly* correlated with the outcome. Nine predictor variables were moderately correlated with the outcome. These variables are: *Enhancement motive to use Tinder*, *Coping motive to use Tinder*, *Social motive to use Tinder, On Tinder looking for committed romantic partner*, *On Tinder looking for uncommitted sex partner*, *Number of online contacts*, *Number of offline contacts*, *Satisfaction with Tinder use*, and *Anxious attachment style*. All these predictors are positively correlated with the outcome, which means that as their values increase, the PTU measure increases as well. Six predictor variables were lowly correlated with the outcome.

Among the 29, some other variables had *r* >.20, which is quite low but non-negligeable correlation. These variables are: *Partner selectiveness on Tinder*, *Dyadic sexual desire*, *Solitary sexual desire*, *Positive urgency impulsivity*, and *Loneliness*. Among them, only *Partner selectiveness on Tinder* was negatively correlated with PTU, which means that as their values increase, the PTU measure decreases.

The ANOVA results evolving the nominal predictor variables indicated that: The effect of participants’ sex on the PTU mean score was significant only for male vs. non-binary and female vs. non-binary individuals (*F*[2, 1384) = 27.95, *p* <.001, η_p_^2^ = 0.039). Indeed, Tukey post-hoc comparisons test showed that female participants’ PTUS mean score was significantly lower than that of non-binary participants (1.77 [SD = 0.63] vs. 1.91 [SD = 0.70]) and that male participants’ PTUS mean score was significantly higher than that of non-binary participants (2.04 [SD = 0.74] vs. 1.91 [SD = 0.70]). There was no significant difference between male and female participants’ PTU mean score (2.04 [SD = 0.74] vs. 1.77 [SD = 0.63]). Also, the effect of the participants’ marital status on the PTUS mean score was not significant (*F*[3, 1383) = 2.233, *p* =.083, η_p_^2^ = 0.005). Similarly, the effect of participants’ sexual orientation on the PTUS score was not significant (*F*[2, 1384) = 0.951, *p* =.387, η_p_^2^ = 0.001). Finally, participants who were looking for Tinder online contacts that can lead to offline contacts had a higher PTUS mean score (2.05, SD = 0.62) than those who were not (1.76, SD = 0.74), *F*(1, 1385) = 62.901, *p* <.001, η_p_^2^ = 0.043)

### Multivariate relationships (predictors’ importance statistics)

Table 3 show the predictor variables in ranking order (machine learning model results). The performance metrics of the machine learning model on the test-set was as follows: R^2^ (percentage of the variance in the outcome that is explained by the predictors) = 58%; MSE (mean squared error) = 0.19.

**Table 3:** Predictors of Tinder problematic use, ranked in decreasing order of importance

Predictors	%IncMSE
Coping motive to use Tinder	30.18
Number of online contacts	24.82
Enhancement motive to use Tinder	21.66
Number of offline contacts	14.41
Social motive to use Tinder	13.89
Number of “Matches”	12.23
Satisfaction with Tinder offline dates	9.94
Anxious attachment style	8.41
On Tinder looking for uncommitted sex partner	7.78
Looking for Tinder online contacts that can lead to offline contacts	7.78
On Tinder looking for committed romantic partner	7.44
Loneliness	7.43
Negative urgency impulsivity	6.47
Number of months using Tinder	5.91
Partner selectiveness on Tinder	5.21
Satisfaction with Tinder use	4.43
Dyadic sexual desire	3.79
Depressive mood	2.90
Positive urgency impulsivity	2.80
Sexual orientation	2.03
Age	1.78
Self-esteem	1.33
Avoidant attachment style	1.08
Lack of perseverance impulsivity	1.06
Lack of premeditation impulsivity	0.77
Sex	0.48
Solitary sexual desire	0.42
Marital status	0.19
Sensation seeking impulsivity	0.13

N = number of participants; %InMSE = per cent increase in mean squared error, a statistical measure indicating the level on the predictor variable importance in the regression machine learning algorithm

As shown in Table 3, among the 29 predictors of PTU, the percent increase in MSE (%IncMSE) ranged from a high of 30.18 (coping motive to use Tinder) to a low of 0.13 (sensation seeking impulsivity), with a median value of 5.21 (partner selectiveness on Tinder). As explained, the more the %IncMSE values is, the more important the variable is for the successful prediction. In other words, the %IncMSE of a given predictor variable reflects the value of the MSE increase in the prediction model if that variable was removed from it. Only the top 6 predictor variables (coping motive to use Tinder; number of online contacts on Tinder; enhancement motive; number of offline contacts; social motive; and number of “Matches”) had %IncMSE scores of over 10. Five predictor variables (the least important for the prediction of PTU) had a %IncMSE scores belowa value of 1. These 5 predictor variables were: lack of premeditation impulsivity; participants’ sex; solitary sexual desire; participants’ marital status; and sensation seeking impulsivity. The 20 strongest predictors of participants’ PTU had a %IncMSE scores above a value of 2. In ranking order, these 20 predictors were: coping motive to use Tinder; number of online contacts on Tinder; enhancement motive; number of offline contacts; social motive; number of “matches”; satisfaction with Tinder offline dates; anxious attachment style; on Tinder looking for uncommitted sex partner; on Tinder looking for online contacts that can lead to offline contacts; on Tinder looking for a committed romantic partner; loneliness; negative urgency impulsivity; number of months using Tinder; level of partner selectiveness on Tinder; satisfaction with Tinder use; dyadic sexual desire; depressive mood; positive urgency impulsivity; and participants’ sexual orientation.

## Discussion

This study aimed to determine, in large sample of Tinder users, the level of problematic dating apps and the most important factors predicting/associated with problematic Tinder use from a set of 29 variables that include socio-demographic characteristics, dispositional traits, and behaviors on the dating app by using a machine learning algorithm.

### PTU Mean score

On the 5-point scale, participants’ mean PTUS score was 1.91 (SD = 0.70). This is below the midpoint of the scale, and, since the standard deviation is relatively small, suggests that most participants were not “addicted” to Tinder. This finding would be in accordance with results from previous studies [7, 8, 17].

### The most important predictors of PTU

The three measured motives for Tinder use (coping [%IncMSE = 30.18], enhancement [%IncMSE = 21.66] and social [%IncMSE = 13.89]) are respectively the first, third and fifth most important predictors of PTU. The higher participants’ scores are on these predictors, the higher the likelihood of PTU. In general, previous studies have shown that the desire to reduce boredom and sociability were among the strongest variables associated with the addictive use of smartphone dating apps [8, 17]. One possible explanation is that some core functionalities of Tinder and similar dating apps, including “swipes,” “likes,” and “matches,” can also allow immediate “feel good” interactions that excite the psychophysiological reward system. The fact that, in the current study, using Tinder as a way to cope with negative emotions was the first (most) important predictor of PTU suggests that individuals in this situation might struggle to find other resources or venues to address distress or other psychological problems. This may lead to Tinder overuse and “dependence” on these interactions for mood boosts and the possibility that tolerance and withdrawal effects may set in.

The second, fourth and sixth most important predictors of participants’ PTU (respectively: the number of online contacts on Tinder [%IncMSE = 24.82], the number of offline contacts [%IncMSE = 14.41] and the number of “matches” [%IncMSE = 12.23]) are related to users’ experience with the app. Likewise, an increase in these predictor values increases the likelihood of PTU. Direct reward of one’s engagement with Tinder might enhance self-perceived desirability [38] and encourage those who experience it to “go on” looking for more contacts and more “matches,” whether as a strategy to maximize their chances to find the “ideal” partner or because they are trapped in a cycle of needing to continuously experience their desirability.

The seventh, nineth, tenth and eleventh most important predictors of PTUS (respectively: satisfaction with Tinder offline dates [%IncMSE **=** 9.94], on Tinder looking for uncommitted sex partner **[**%IncMSE **=** 7.79], on Tinder looking for online contacts that can lead to offline contacts [%IncMSE **=** 7.78] and on Tinder looking for committed romantic partner [%IncMSE = 7.44]) are also related to users’ behavior on, or experience with, the app. Higher values in these predictors (except for on Tinder looking for uncommitted sex partner) increase the likelihood of PTU. It is interesting that the search for “serious” and romantic relationships and looking for uncommitted sex partner are positively associated with PTU. These results are in accordance with some previous studies that showed that participants searching for “true love” and those looking for casual sex were significantly more likely to experience problematic use [8, 17].

The eighth, twelfth and thirteenth most important predictor of PTU (respectively: anxious attachment style [%IncMSE = 8.41], loneliness [%IncMSE = 7.43] and negative urgency impulsivity [%IncMSE = 6.47]) seem to relate to participants’ psychological functioning. Higher values on these predictors are associated with greater PTU. Previous studies have shown that insecure attachment styles are associated with more addictive Tinder use as well as the negative urgency trait [19]. The neuroticism personality trait, in particular, seems related to overuse of online dating services [39]. Individuals scoring higher on neuroticism are also more likely to experience high levels of impulsivity and anxious attachment style [40, 41] and have a higher likelihood of developing internet and other forms of addiction compared with those who have low neuroticism scores [39]. Corroborating these results, negative urgency—an impulsivity trait strongly associated with neuroticism [42]—has been associated with poor inhibitory control and decision making [43]. Finally, loneliness might also be linked to the boredom motive of engaging with Tinder, found be a significant predictor of PTU in previous studies [8].

The remaining predictor variables have a %IncMSE score below 6, which suggests that they are relatively less important in predicting participants’ PTU.

Notably, none of the 4 sociodemographic variables included in the current study (age, sex, marital status, sexual orientation) were ranked as important predictors of PTU. This finding confirms data from some Tinder studies but differs from others that linked being male, younger and gay or bisexual to addictive use of online dating services [5, 6, 9, 17].

Dyadic and solitary sexual desire also seem to have a relatively week relationship with addictive Tinder use. Sexual desire, especially the dyadic type, probably does contribute to Tinder use, but only marginally to addictive use. This could be due to the fact that addictive use is more strongly maintained by coping mechanism intended to regulate affect than by gratification seeking mechanisms. This is suggested by the weight of coping motives in the present study and has also been reported elsewhere [44].

Finally, the current study results suggest that three predictor variables (age, the level of partner selectiveness on Tinder and satisfaction with Tinder online date) are negatively associated with participants’ PTU, with higher ages and scores being associated with less PTU. The older users are, the less likely they are to experience PTU. Previous studies [8, 17] have shown a similar relationship between age and PTU. One possible explanation is that older individuals are more likely to be in committed relationships and to have familial and professional responsibilities [23], potentially limiting their availability for heavy Tinder use. High level of partner selectiveness may be an indication that the user engaged on Tinder with a “serious” goal and a relatively significant level of self-control, which in turn may lead to low levels of PTU. The finding that satisfaction with Tinder offline dates is negatively associated with PTU might reflect the fact that once a satisfying relationship is established with a person found on Tinder, the user may feel no ongoing need for the platform.

### Limitations

As participants were not recruited randomly, the extent to which the present study sample is representative of overall Tinder users is unknown. The extent to which the data apply to users of other dating apps, some of which cater to sexual minorities or individuals seeking a particular profile, is similarly unknown. Also, self-selection bias cannot be ruled out [45]. Further, the study has a cross sectional design and therefore cannot assess longitudinal interactions among variables.

Other factors that could be associated with addictive online dating, such as level of education, occupation, comfort level with new technologies, other personality traits, substance use disorders and other behavioral addictions (e.g., sex “addiction”, problematic pornography use, internet gaming disorder), were not assessed and would have painted a more complete picture and enhanced the interpretability of our findings. Still, our choice of the factors studied was driven by the desire to explore ones that haven’t received adequate research attention, while keeping the number of variables queried relatively contained to avoid survey fatigue.

Finally, the machine learning statistical model included a relatively large number of covariates (29). In multivariate statistical models, as the number of dimensions increases, the risk of multicollinearity increases, and it becomes easier to confuse noise for real correlations [46, 47]. However, in the present study, we tried to minimize this risk by using a machine learning algorithm (Random Forest) that was designed to include a considerable number of features and handle multicollinearity [21].

## Conclusion

In summary, the findings suggest that the ‘addictive’ use of dating apps is strongly related to the motives driving the user to the app in the first place. Overall, participants’ level of PTU appears to be a function of the interaction between their experience on the app (e.g., number of contacts, number of “matches”, level of satisfaction) and their dispositional and situational characteristics. This is in accordance with the I-PACE model of Internet use disorder [22]. In particular, psychological problems, boredom and loneliness appear to drive PTU in many individuals, and these are problems that an online dating platform cannot resolve on its own. This suggests the need for services that can identify these issues in users of online dating services and address them in a way that Tinder and similar apps are not designed to. Finally, access to a large reservoir of potential partners means that, for some individuals, even if they find a quality match, they are tempted by the prospect of finding an even better one and therefore will continue to search indefinitely in a way that can appear addictive [10]. As with gambling, a significant win often triggers the desire for an even bigger one [48].

## Data Availability

The material used in this study and data supporting this finding are available at: https://gitlab.huma-num.fr/gveracruz/dating-apps-problematic-use/-/tree/main/Data.

## Footnotes

1 Given that there is persistent debate regarding whether these maladaptive online behaviors constitute addictive disorders or are manifestations of other established psychopathologies and that such terminology is not recognized in the DSM-5, in the present study, we used the expression “problematic use” to mean an addiction-like use of the studied digital service (online dating, dating app, Tinder). The components of such “problematic use” are presented in the Methods section below (see “The Study Outcome” sub-section).

## Abbreviations

PTU: Problematic Tinder use
PTUS: Problematic Tinder Use Scale
SDHS: Short Happiness and Depression Scale
SISE: Single Item Self-Esteem
CMQ: Cybersex Motives Questionnaire
ECR-R: Experience in Close Relationship– Revised
SDI: Sexual Desire Inventory
CIUS: Compulsive Internet Use Scale
UPPS-P: Urgency, Premeditation (lack of), Perseverance (lack of), Sensation Seeking, Positive Urgency, Impulsive Behavior Scale
M: Mean
SD: Standard deviation
FR: Random Forest
%IncMSE: Per cent of mean squared error
MSE: Mean squared error
ANOVA: Analysis of variance
